# Overall and Site‐Specific Cancer Mortality Among Older Migrants and Nonmigrants in Finland: A Population Register Study on All Deaths, 2002–2020

**DOI:** 10.1002/cam4.71380

**Published:** 2025-11-23

**Authors:** Laura Kemppainen, Teemu Kemppainen, Jani Raitanen, Linda Enroth, Pauliina Halonen, Marja Jylhä, Anne Kouvonen, Jutta Pulkki

**Affiliations:** ^1^ Faculty of Social Sciences, University of Helsinki Helsinki Finland; ^2^ Faculty of Social Sciences (Health Sciences) and Gerontology Research Center (GEREC), Tampere University Tampere Finland; ^3^ Department of Geosciences and Geography University of Helsinki Helsinki Finland; ^4^ The UKK Institute for Health Promotion Research Tampere Finland; ^5^ Department of Healthcare and Social Welfare, Services Unit Finnish Institute for Health and Welfare Helsinki Finland; ^6^ Centre for Public Health Queen's University Belfast Belfast UK

**Keywords:** cancer risk factors, epidemiology, statistical methods

## Abstract

**Objective:**

This study examined all‐cause and cause‐specific cancer mortality among older migrants and non‐migrants in Finland and the role of socioeconomic status in mortality differences.

**Methods:**

We used the Finnish Causes of Death Register on all deaths (2002–2020) among individuals aged ≥ 70 (*N* = 718,717) and the corresponding population‐at‐risk data (*N* = 13,241,620 person years). Poisson regression was used with two sequential models adjusting for age at death, calendar year, and region of residence in Finland (M1), and personal annual disposable income (M2).

**Results:**

We found an overall cancer mortality advantage for both migrant men (IRR in the full model 0.83, 95% CI: 0.78–0.89) and migrant women (IRR: 0.89, 95% CI: 0.83–0.95) and lung cancer mortality advantage for migrant men (IRR: 0.77, 95% CI: 0.67–0.89) and women (IRR: 0.67, 95% CI: 0.53–0.85). For migrant men, advantage was found in pancreatic cancer (IRR: 0.76, 95% CI: 0.58–0.99), prostate cancer (IRR: 0.78, 95% CI: 0.66–0.93), and leukaemia and lymphoma (IRR: 0.73, 95% CI: 0.58–0.93), and for women in genital cancers (IRR: 0.69, 95% CI: 0.55–0.86). Notable variations were observed by region of origin and in certain cases, migrants' lower income obscured the full extent of their cancer mortality advantage. A mortality disadvantage was observed in stomach cancer among men (IRR: 2.76, 95% CI: 2.08–3.65) and women (IRR: 2.32, 95% CI: 1.79–3.00) born in the former USSR. Liver cancer mortality disadvantage was found for men from the Global South and East (IRR: 2.00, 95% CI: 1.10–3.61), and this association was attenuated after adjustment for personal disposable income. In cancers of the urinary tract, men born in Sweden had elevated mortality (IRR: 2.09, 95% CI: 1.14–3.81).

**Conclusion:**

Finings underscore the need for targeted cancer prevention and screening programmes that account for the diverse backgrounds, sex, socioeconomic status, and health risks of migrant populations, particularly those from higher‐risk regions.

## 1 Introduction

With increased longevity, the number of older individuals is expected to rise significantly over the coming decades. As cancer risk increases with age, nearly half of new cancer cases globally are diagnosed in adults aged 65 and over [1]. To address the upcoming challenges in cancer care, more research is needed on the cancer burden among the oldest people [2, 3]. At the same time, the number of international migrants is rising, yet the intersection of aging and migration—older migrants—remains understudied, particularly in the context of mortality patterns [4, 5]. This gap in research is concerning, as older migrants may face unique health challenges and barriers to accessing care, which could influence their cancer outcomes and mortality [6].

Research from high‐income countries shows that while migrants exhibit a general mortality advantage in many disease categories, including cancers [7], this advantage follows a U‐shaped age association, with mortality advantage most pronounced among younger adults and tapering off from around age 40 [8]. This tapering may be attributed to a diminishing “healthy migrant effect” as initial health‐based selection advantages and beneficial lifestyle patterns wane over time [8, 9]. While some research suggests that data artifacts or selective return migration (“salmon bias”) might explain lower migrant mortality [9, 10], recent studies do not fully support these explanations [8, 11]. Among the oldest age groups, migrant mortality advantage varies, potentially being maintained, reduced, or reversed depending on the migrant group and destination country [8]. Further research is needed to clarify the mortality advantage and its mechanisms among older migrants.

A global review on migrant mortality [7] has found an overall cancer mortality advantage for migrants in high‐income countries, particularly notable for cancers of the urinary tract, neurological system, respiratory system, bone, and female genital organs. Research on migrants from non‐Western countries to Europe has found that they were more prone to cancers linked to early‐life infections, such as liver, cervical, and stomach cancers, and less likely to develop cancers associated with a Western lifestyle, such as colorectal, breast, and prostate cancers [12, 13, 14]. Migrants face multiple barriers to accessing healthcare and are less likely to be diagnosed at an early stage, which is often associated with poorer cancer survival [15]. However, the evidence remains mixed and varies by country of origin, destination country, and cancer site, with some studies indicating better survival among migrants for specific cancers compared to locally born people [16, 17, 18]. Migrants tend to live longer, even though they experience higher morbidity, a pattern known as the migrant morbidity‐mortality paradox [19].

In our previous research on all‐cause and cause‐specific mortality, we identified a lower overall cancer mortality among migrants aged 70 and older, in both men and women, compared to the Finnish‐born population [20]. This advantage in cancer mortality varied by region of origin, with the strongest effect observed among migrants from the Global South and East. However, our prior study did not examine cancer‐site‐specific mortality differences, as overall cancer mortality was analyzed as one of multiple causes of death. An earlier study from Belgium [4], focusing on cause‐specific mortality among older migrants (aged 50 and above) based on 2009 data, similarly found that older migrants generally had lower cancer mortality compared to locally born peers, though this varied by sex, cancer site, and migrant group.

Recent reports highlight significant disparities in overall cancer mortality across socio‐economic groups in EU countries, especially among men [21]. Older migrants are often at heightened risk of low socioeconomic status (SES), such as low income [5, 22], which can affect both their risk for developing cancers and their survival [23]. Lower SES is associated with certain cancers such as lung cancer, while higher SES is associated, for example, with a higher risk of breast and prostate cancers [13, 24]. Higher SES is also associated with lower case fatality [25]. However, socioeconomic differences in cancer risk and mortality vary globally, partly reflecting the varying SES differences in lifestyles [26]. People with lower SES and from migrant backgrounds participate less frequently in cancer screening programs, which can affect survival [21, 27]. Moreover, the underlying processes of the SES gradient remain unclear among migrants [28], highlighting the need for further research on how SES influences cancer mortality in different populations.

This study examines disparities in cancer mortality among adults aged 70 years and older in Finland, comparing migrant and non‐migrant populations. We aim to identify migrant subgroups with higher or lower cancer mortality compared to the Finnish‐born population, and to assess the role of income differences in these disparities. Analyzes are conducted separately for men and women, controlling for age at death, calendar year, region of residence and personal annual disposable income.

### 1.1 Context of the Study

The context of this study is Finland, a Nordic welfare state with universal and relatively low‐cost access to health care for all residents [29]. Finland has seen increasing immigration in recent decades. In 2024 there were 600,000 persons speaking a foreign language as their first language [30]. In the capital region, the share of foreign‐language speakers is approximately 25%, compared to around 11% in Finland overall [31]. The amount of foreign‐language speakers is projected to increase especially in the capital region, with particularly rapid relative growth expected among the working‐age and older people [32].

Estimated cancer incidence in Finland is near the European Union average but cancer mortality rates are among the lowest in the EU [33]. The most common new cancer cases in the oldest age groups (≥ 75) are breast cancer for women and prostate cancer for men [34]. Other common cancers among older people are colorectal cancers, lymphomas and leukemia, and lung cancer [34]. Cancer incidence is higher among people with lower education levels [33].

Compared to other EU countries, Finland shows higher levels of overweight and occupational chemical exposure as cancer risk factors, but lower than EU average levels of other major risk factors, including smoking, alcohol use, physical inactivity, and air pollution [33]. A comparative research report on lung cancer care indicated shortcomings in the Finnish cancer treatment compared to other Nordic countries, including limited access to diagnostic imaging in primary care, long waiting times, and initial patient appointments being more often with nurses than doctors, all of which may contribute to delayed diagnoses, later‐stage detection, and lower survival outcomes [35].

## 2 Materials and Methods

This is a population‐based register study. The data include all deaths at age 70 or older in Finland in 2002–2020 (*N* = 718,717) drawn from the Cause of Death Register (Statistics Finland) and the corresponding risk population (*N* = 13,241,620 person years) from the Statistics Finland Population Structure. Of the recorded deaths, 1.1% (*n* = 7665), and of the risk population 1.3% (*n* = 175,626) were migrants. Migrant was defined as having been born outside of Finland. Information on the age at migration or length of stay was not available in the register, and thus, cannot be accounted for in our analyzes.

The Finnish Cause of Death register is generally regarded as accurate and robust for epidemiological research [36]. The proportion of so‐called “garbage codes” and ill‐defined causes of death (ICD codes R00–R99) is low [37, 38]. However, migrants are overrepresented in the ill‐defined category, mainly because many of these deaths occurred outside Finland and the body had not been brought to Finland [20, 39].

The underlying causes of death were obtained from the Finnish Causes of Death Register. In the register, underlying cause of death refers to “the disease which has initiated the series of illnesses leading directly to death, or the circumstances connected with an accident or an act of violence which caused the injury or poisoning leading to death” [40]. Our selection of cancer sites for inclusion was guided primarily by the availability of sufficient case numbers in most subgroups, defined as more than five cases per subgroup, and secondarily by comparability with existing studies on cancer mortality in Europe [23], among migrants [12, 14] and among older adults [2, 3]. Some sites, such as melanomas (C43–C44), brain and CNS cancers (C69–C72), and thyroid and other endocrine cancers (C73–C75), were excluded due to limited numbers of cases among migrants. Prostate cancer (C61) and lung cancer (C34) were the leading causes of death in their respective broader categories and were therefore analyzed separately. Stomach cancer (C16) was included because of its relevance for the former Union of Soviet Socialist Republics (USSR) subgroup, even though case numbers were low in other subgroups. Ovarian, cervical, and other female reproductive cancers (C51–C58) were analyzed together under the broader category of “female genital cancers”. Similarly, cancers of the urinary tract (C64–C68) as well as lymphoma and leukemia (C81–C96) were examined as broader categories. We were able to provide reliable evidence for the following cancer death categories (ICD‐10 codes):

– All cancer deaths (C00–D48)
– Stomach cancer (C16)
– Colorectal cancer (C18–C20)
– Liver cancer (C22)
– Pancreatic cancer (C25)
– Lung cancer (C34)
– Cancers of the urinary tract (C64–C68)
– Lymphoma and leukemia (C81–C96)
– Breast cancer (C50) for women
– Genital cancers (C51–C58) for women
– Prostate cancer (C61) for men

We also obtained the following register information from Statistics Finland:

- Calendar year (2002–2020)
- Age at death (70–78, 79–84, 85–89, ≥ 90 years) (the age group categories correspond to the age quartiles of the migrant population in the causes of death register)
- Sex (women, men)
- Personal annual disposable income (≤ 11,999, 11,200–14,399, 14,400–18,399, ≥ 18,400 EUR)
- Region of residence in Finland (Uusimaa, Southern Finland, Northern and Eastern Finland, Western Finland, Åland)
- Country of birth, categorized by geographical region of origin (Finland, Former USSR, Sweden, Eastern Europe and the Former Yugoslavia, other Global North and West, and the Global South and East) (see Figure S1)

**FIGURE 1:**
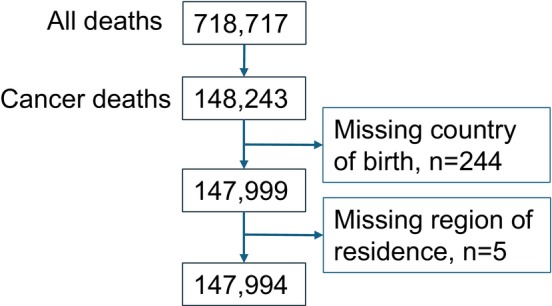
Flowchart of missing data.

Cancer deaths (ICD: C00–D48) comprised 21% (*n* = 148,243) of all deaths at age ≥ 70 in Finland in 2002–2020. These included 244 persons (0.2% of cancer deaths) with an unknown country of birth, which were excluded from the analytical sample resulting in 147,999 cases. The missing information on income (*n* = 767 in all deaths) was imputed using regression predictions. Our imputation model used the following predicting variables: interaction of age at death (10 categories) and sex, region of residence in Finland, region of birth and the calendar year. The residence variable had 93 missing observations, which largely overlapped with missing country of birth data. After excluding cases with missing values in country of birth, the missingness was five observations in the region of residence and income variables (Figure 1).

To calculate mortality rates, we obtained the corresponding risk population sizes for all combinations of the mentioned variables (data available by request from Statistics Finland). As the risk population reflected the situation on the last day of a year, to obtain an estimate of the true risk population we added the number of all deaths per year to the end‐of‐year risk population, resulting in 13,960,337 person years (see Table S1).

**FIGURE 2:**
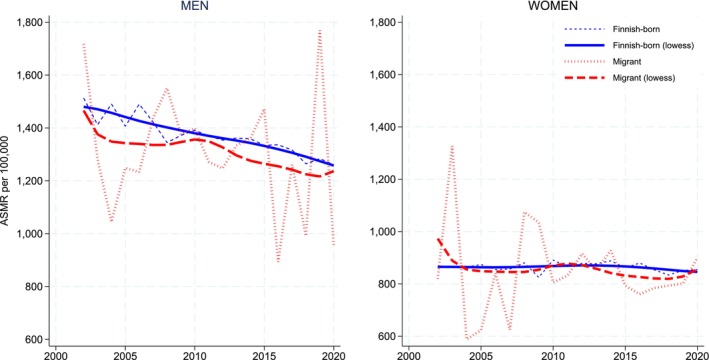
Age‐standardized mortality rates (ASMRs) for cancer per 100,000 persons with locally weighted scatterplot smoothing (LOWESS) curve, by calendar year, among Finnish‐born and migrant populations for women and men. Standardized to the Finnish‐born population aged ≥ 70 years.

Overall cancer mortality differences were first analyzed as crude death rates and by direct age‐standardized mortality rates (ASMRs) and their 95% confidence intervals (CIs) for each category of region of origin. The Finnish‐born population aged ≥ 70 years was used as the standard population. For the analysis of changes over time, we calculated ASMRs annually from 2002 to 2020 for migrants and those born in Finland separately for men and women (Figure 2). Since the numbers for migrants were rather small and showed considerable fluctuation, we applied locally weighted scatterplot smoothing (LOWESS), a local regression method that fits weighted regressions to small subsets of data to reveal underlying trends more clearly [41, 42].

Incidence rate ratios (IRRs) and their 95% confidence intervals (CIs) from Poisson regression were used to study relative mortality differences in overall and site‐specific cancer by region of origin, using the Finnish‐born population as the reference category. Poisson regression has been shown to provide consistent coefficient and interval estimates also in those situations that are often thought to require alternative estimators, such as the choice of negative binomial, when overdispersion is present [43, 44]. Robust standard errors (Huber‐White/sandwich) were estimated. To further examine the association of income in mortality differences, two models were specified: Model 1 included age at death, calendar year, and the region of residence in Finland as explanatory variables, while Model 2 also added personal annual disposable income.

Analyses were performed with Stata version 17 (StataCorp, College Station, Texas).

## 3 Results

A descriptive overview of the study population reveals demographic and socio‐economic differences based on geographical origin. The proportion of women varies by geographical origin, ranging from 42% in the Global North and West to 71% in the Former USSR. The youngest age group is notably prevalent among individuals from Sweden (73%) and the Global South and East (73%). Socio‐economic disparities emerge in personal annual disposable income data: while individuals with a Swedish background are relatively affluent (48% in the highest income quartile), the opposite holds for those from the Former USSR, with 57% in the lowest income quartile. Geographically, migrant populations tend to be concentrated in the Helsinki‐Uusimaa region, particularly those from the Global South and East (62%). Full descriptive statistics for the study population are presented in Table S1.

Table 1 presents crude and age‐standardized mortality rates (ASMRs) for overall cancer mortality for men and women. Crude death rates were lower among migrants in general compared to the Finnish‐born population. Among subgroups, crude death rates were higher among men and women born in Sweden, men from the Former USSR countries and women from Eastern Europe and Former Yugoslavia compared to the Finnish‐born population. Age adjustment attenuated the differences, but only a few were statistically significant. The all‐cancer mortality advantage was statistically significant for men and women born in the Global North and West and the Global South and East.

**TABLE 1:** Cancer deaths, crude and age‐standardized mortality rates (ASMRs, 95% confidence intervals (CIs)) for women and men aged ≥ 70 years in Finland by region of origin.

	Risk population	Cancer deaths	Crude death rate	ASMR	95% confidence intervals	
Men						
Finnish‐born	5,499,037	74,852	1361	1361	1352	1371
All migrants	68,982	852	1235	1287	1201	1374
Former USSR	30,466	435	1428	1428	1295	1561
Sweden	6235	87	1395	1681	1308	2055
Eastern Europe and Former Yugoslavia	4233	41	969	1119	767	1470
Global North and West	18,340	205	1118	1156	998	1314
Global South and East	9708	84	865	962	743	1181
Women						
Finnish‐born	8,268,360	71,410	864	864	857	870
All migrants	106,644	885	830	842	787	898
Former USSR	74,318	638	859	864	797	931
Sweden	6220	60	965	1024	754	1295
Eastern Europe and Former Yugoslavia	4384	39	890	1046	700	1393
Global North and West	13,159	103	783	708	568	847
Global South and East	8563	45	526	586	405	768

*Note:* Rates per 100,000; standardized to the Finnish‐born population aged ≥ 70.

Moving on to time trends, Figure 2 illustrates age‐standardized cancer mortality rate trends over the study period (2002–2020). The general trend shows rather stable cancer death rates for women, while men's cancer death rates have steadily declined over the years. When comparing Finnish‐born and migrant populations, migrants exhibit lower ASMRs, particularly among men.

**FIGURE 3:**
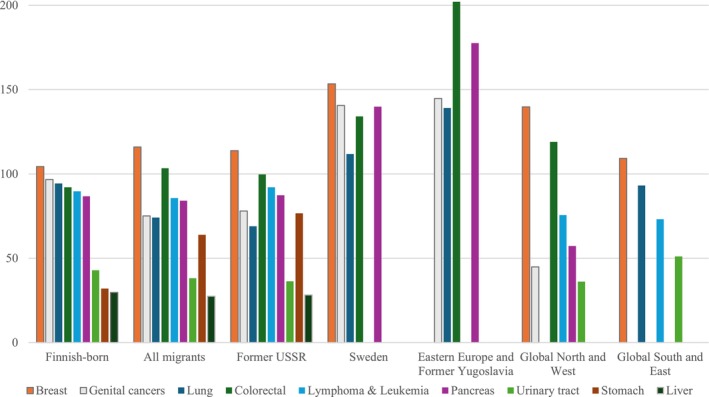
Age‐standardized mortality rates per 100,000 persons by region of origin and cancer site for women. Standardized to the Finnish‐born population aged ≥ 70. Only cancer categories with ≥ 5 deaths per subgroup are shown.

**FIGURE 4:**
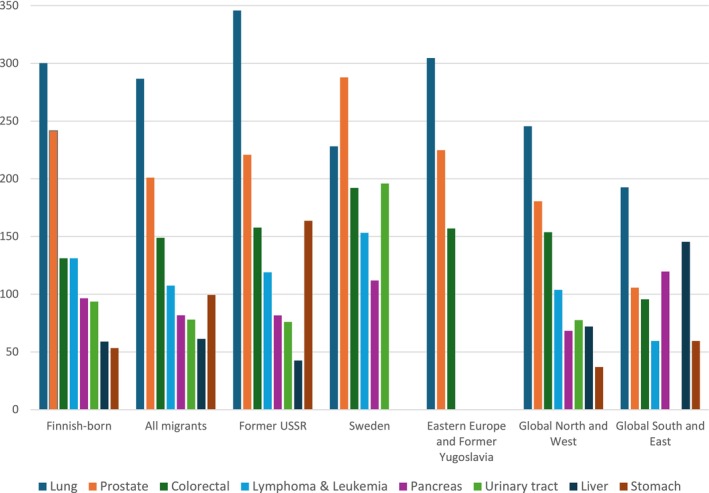
Age‐standardized mortality rates per 100,000 persons by region of origin and cancer site for men. Standardized to the Finnish‐born population aged ≥ 70. Only cancer categories with ≥ 5 deaths per subgroup are shown.

Figures 3 and 4 show age‐standardized cancer mortality rates (2002–2020) in each studied cancer site by region of origin for women and men. Only cancer categories with at least five deaths in a given subgroup were included to ensure stable estimates. It should be noted that the figures for all migrant groups are heavily influenced by the Former USSR subgroup, which is the largest migrant group in Finland.

Compared to Finnish‐born women, migrant women had higher mortality for breast (116 per 100,000 persons vs. 104 of Finnish‐born), colorectal (102 vs. 92), and stomach cancer (64 vs. 32), and lower mortality for genital (75 vs. 97) and lung cancer (74 vs. 94) (Figure 3). Among the subgroups, women from Sweden as well as those from Eastern Europe and the former Yugoslavia, exhibited elevated mortality across all analyzed cancer sites. Women from other countries of the Global North and West showed elevated mortality for breast (140) and colorectal (119) cancer, but lower mortality for genital (45) and pancreas (57) cancers compared to other groups.

Among men (Figure 4), compared to Finnish‐born men, migrants exhibited lower mortality for prostate cancer (201 vs. 242) but higher mortality for colorectal (149 vs. 131) and stomach (99 vs. 53) cancers. Men from the Former USSR showed higher mortality for lung (346) and stomach (164) cancers compared to Finnish‐born men (300 and 53, respectively). Except for lung cancer (228), men from Sweden had elevated mortality across most cancer sites, with higher rates for urinary tract (196) and colorectal (192) cancers compared to other subgroups. Men from Eastern Europe and former Yugoslavia had elevated mortality rates for colorectal (157) and lung (305) cancer. Men from other countries of the Global North and West generally had lower mortality than other subgroups across cancer sites, except for colorectal cancer (154) compared to Finnish‐born men (131) and men from the Global South and East (96). Men from the Global South and East had lower mortality overall, except for liver (146) and pancreatic (120) cancer.

The underlying causes of death were obtained from the Finnish Causes of Death Register. In the register, underlying cause of death refers to “the disease which has initiated the series of illnesses leading directly to death, or the circumstances connected with an accident or an act of violence which caused the injury or poisoning leading to death” (Table 2) presents incidence rate ratios (IRR) and 95% confidence intervals (CI) from the Poisson regression models for women. For overall cancer mortality in women, Model 1, adjusted for age at death, calendar year, and region of residence in Finland, shows a mortality advantage for migrants from the Global South and East (IRR: 0.65, 95% CI: 0.49–0.88). Model 2 adds personal annual disposable income to Model 1, revealing a statistically significant mortality advantage for women from the former USSR (IRR: 0.90, 95% CI: 0.84–0.98). Because of this, the general migrant advantage became statistically significant (IRR: 0.89, 95% CI: 0.83–0.95). Regarding women from the Global South and East, the result became somewhat more pronounced in the model that adjusts for income (IRR: 0.60, 95% CI: 0.44–0.81). Women born in the Global North and West exhibited a tendency toward a mortality advantage in both models (*p* = 0.052) (Table 2).

**TABLE 2:** Incidence rate ratios (IRR) and 95% confidence intervals (CI) showing relative mortality differences for women from the Poisson regression model.

	Model 1				Model 2		
	Cases (*n*)	IRR	95% CI		IRR	95% CI	
All cancers							
Finnish‐born	71,410	Ref.	.	.	.	.	.
All migrants	885	0.95	0.89	1.02	**0.89**	**0.83**	**0.95**
Former USSR	638	0.98	0.90	1.06	**0.90**	**0.84**	**0.98**
Sweden	60	1.19	0.93	1.51	1.20	0.94	1.53
Eastern Europe and Former Yugoslavia	39	1.12	0.82	1.53	1.04	0.77	1.42
Global North and West	103	0.83	0.68	1.01	0.82	0.68	1.00
Global South and East	45	**0.66**	**0.49**	**0.88**	**0.60**	**0.44**	**0.81**
Lung							
Finnish‐born	7794	Ref.	.	.	.	.	.
All migrants	79	**0.69**	**0.55**	**0.87**	**0.67**	**0.53**	**0.85**
Former USSR	51	**0.64**	**0.48**	**0.85**	**0.62**	**0.46**	**0.82**
Sweden	9	1.34	0.71	2.55	1.37	0.72	2.59
Eastern Europe and Former Yugoslavia	6	1.30	0.59	2.89	1.27	0.57	2.81
Global North and West	< 5	.	.	.	.	.	.
Global South and East	9	0.90	0.44	1.84	0.86	0.42	1.74
Colorectal							
Finnish‐born	7619	Ref.	.	.	.	.	.
All migrants	108	1.11	0.92	1.34	1.04	0.86	1.26
Former USSR	73	1.07	0.85	1.34	0.99	0.79	1.24
Sweden	7	1.33	0.63	2.78	1.34	0.64	2.81
Eastern Europe and Former Yugoslavia	7	1.99	0.95	4.16	1.85	0.89	3.85
Global North and West	17	1.25	0.78	2.00	1.23	0.77	1.98
Global South and East	< 5	.	.	.	.	.	.
Stomach							
Finnish‐born	2647	Ref.	.	.	.	.	.
All migrants	67	**2.14**	**1.69**	**2.73**	**1.95**	**1.53**	**2.48**
Former USSR	57	**2.60**	**2.01**	**3.36**	**2.32**	**1.79**	**3.00**
Sweden	< 5	.	.	.	.	.	.
Eastern Europe and Former Yugoslavia	< 5	.	.	.	.	.	.
Global North and West	< 5	.	.	.	.	.	.
Global South and East	< 5	.	.	.	.	.	.
Pancreas							
Finnish‐born	7170	Ref.	.	.	.	.	.
All migrants	89	0.94	0.77	1.15	0.88	0.72	1.09
Former USSR	65	0.98	0.77	1.24	0.91	0.72	1.15
Sweden	8	1.94	0.78	3.13	1.58	0.79	3.15
Eastern Europe and Former Yugoslavia	7	1.94	0.93	4.05	1.80	0.86	3.78
Global North and West	8	0.66	0.33	1.32	0.65	0.33	1.30
Global South and East	< 5	.	.	.	.	.	.
Liver							
Finnish‐born	3247	Ref.	.	.	.	.	.
All migrants	29	0.92	0.63	1.33	0.82	056	1.19
Former USSR	21	0.94	0.62	1.43	0.82	0.53	1.25
Sweden	< 5	.	.	.	.	.	.
Eastern Europe and Former Yugoslavia	< 5	.	.	.	.	.	.
Global North and West	< 5	.	.	.	.	.	.
Global South and East	< 5	.	.	.	.	.	.
Urinary tract							
Finnish‐born	3544	Ref.	.	.	.	.	.
All migrants	40	0.89	0.66	1.21	0.82	0.60	1.11
Former USSR	27	0.85	0.59	1.23	0.86	0.22	3.42
Sweden	< 5	.	.	.	.	.	.
Eastern Europe and Former Yugoslavia	< 5	.	.	.	.	.	.
Global North and West	6	0.96	0.43	2.14	0.96	0.43	2.13
Global South and East	5	1.64	0.69	3.87	1.44	0.61	3.42
Breast							
Finnish‐born	8620	Ref.	.	.	.	.	.
All migrants	122	1.05	0.88	1.26	1.04	0.87	1.24
Former USSR	84	1.03	0.83	1.28	1.02	0.82	1.26
Sweden	10	1.61	0.86	2.98	1.61	0.86	2.98
Eastern Europe and Former Yugoslavia	< 5	.	.	.	.	.	.
Global North and West	20	1.33	0.86	2.05	1.32	0.85	2.04
Global South and East	6	0.67	0.30	1.49	0.66	0.29	1.46
Genital cancers							
Finnish‐born	7998	Ref.	.	.	.	.	.
All migrants	79	**0.75**	**0.60**	**0.93**	**0.69**	**0.55**	**0.86**
Former USSR	58	0.79	0.61	1.02	**0.71**	**0.55**	**0.92**
Sweden	7	1.20	0.58	2.52	1.21	0.58	2.53
Eastern Europe and Former Yugoslavia	5	1.21	0.51	2.90	1.10	0.46	2.63
Global North and West	7	0.52	0.25	1.09	0.51	0.24	1.07
Global South and East	< 5	.	.	.	.	.	.
Lymphoma and leukemia							
Finnish‐born	7416	Ref.	.	.	.	.	.
All migrants	90	0.94	0.76	1.15	0.88	0.71	1.08
Former USSR	68	1.00	0.79	1.27	0.93	0.73	1.18
Sweden	< 5	.	.	.	.	.	.
Eastern Europe and Former Yugoslavia	< 5	.	.	.	.	.	.
Global North and West	11	0.86	0.48	1.55	0.85	0.47	1.54
Global South and East	5	0.72	0.30	1.74	0.66	0.27	1.60

*Note:* Model 1: Age at death, calendar year, region of residence in Finland; Model 2: Model 1 + annual personal disposable income. Statistically significant IRRs in bold (*p* ≤ 0.05). “.” indicates < 5 observations.

In lung cancer, women from the Former USSR exhibited a mortality advantage that did not change with income adjustment (Model 1, IRR: 0.64, 95% CI: 0.48–0.85/Model 2, IRR: 0.62, 95% CI: 0.46–0.82) contributing to the mortality advantage in the entire migrant group. The mortality disadvantage in ASMRs that was observed for women from Sweden, Eastern Europe and Yugoslavia in Figure 2 did not reach statistical significance in Poisson regression analysis.

For stomach cancer, the sample size was large enough for analysis only for migrants from the USSR, who showed a clear mortality disadvantage compared to Finnish‐born women in both models (Model 1, IRR: 2.60, 95% CI: 2.01–3.36; Model 2, IRR: 2.32, 95% CI: 1.79–3.00). Here, income adjustment somewhat attenuated the difference.

In the category of female genital cancers, migrant women showed a mortality advantage in both models (Model 1, IRR: 0.75, 95% CI: 0.60–0.93; Model 2, IRR: 0.69, 95% CI: 0.55–0.86). However, for women from the Former USSR the mortality advantage became significant only after adjusting for income in Model 2 (Model 1, IRR: 0.79, 95% CI: 0.61–1.02; Model 2, IRR: 0.71, 95% CI: 0.55–0.92).

In colorectal, pancreatic, liver, urinary tract, breast cancer, and lymphomas and leukemia, no statistically significant differences were found between the groups of women.

Table 3 shows the incidence rate ratios (IRRs) from Poisson regression for men. In Model 1, an overall cancer mortality advantage was found for men from both the Global North and West (IRR: 0.84, 95% CI: 0.73–0.96) and the Global South and East (IRR: 0.72, 95% CI: 0.58–0.90). After adjusting for income (Model 2), the mortality advantage became stronger and statistically significant for all male migrant groups, except for men from Sweden, with IRRs ranging from 0.61 (Global South and East, 95% CI: 0.49–0.75) to 0.89 (Former USSR, 95% CI: 0.81–0.97).

**TABLE 3:** Incidence rate ratios (IRRs) and 95% confidence intervals (CIs) showing relative mortality differences for men from the Poisson regression models.

	Cases (*n*)	Model 1			Model 2		
		IRR	95% CI		IRR	95% CI	
All cancers							
Finnish‐born	74,852	ref.	.	.	.	.	.
All migrants	852	0.94	0.87	1.01	**0.83**	**0.78**	**0.89**
Former USSR	435	1.04	0.95	1.15	**0.89**	**0.81**	**0.97**
Sweden	87	1.11	0.89	1.39	1.14	0.91	1.42
Eastern Europe and Former Yugoslavia	41	0.78	0.58	1.04	**0.69**	**0.52**	**0.93**
Global North and West	205	**0.84**	**0.73**	**0.96**	**0.80**	**0.69**	**0.92**
Global South and East	84	**0.72**	**0.58**	**0.90**	**0.61**	**0.49**	**0.75**
Lung							
Finnish‐born	16,507	ref.	.	.	.	.	.
All migrants	194	0.97	0.83	1.12	**0.77**	**0.67**	**0.89**
Former USSR	106	1.15	0.95	1.39	0.85	0.70	1.03
Sweden	14	0.85	0.48	1.52	0.89	0.50	1.60
Eastern Europe and Former Yugoslavia	13	1.10	0.65	1.85	0.88	0.52	1.49
Global North and West	44	0.82	0.61	1.11	0.74	0.55	1.01
Global South and East	17	0.64	0.39	1.03	**0.46**	**0.28**	**0.75**
Colorectal							
Finnish‐born	7210	ref.	.	.	.	.	.
All migrants	98	1.10	0.90	1.34	1.00	0.82	1.22
Former USSR	48	1.18	0.90	1.54	1.03	0.78	1.35
Sweden	10	1.31	0.71	2.42	1.33	0.72	2.46
Eastern Europe and Former Yugoslavia	5	0.96	0.40	2.31	0.87	0.36	2.10
Global North and West	27	1.13	0.77	1.66	1.08	0.74	1.59
Global South and East	8	0.69	0.35	1.35	0.60	0.30	1.18
Stomach							
Finnish‐born	2931	ref.	.	.	.	.	.
All migrants	67	**2.11**	**1.66**	**2.70**	**1.84**	**1.44**	**2.36**
Former USSR	50	**3.32**	**2.52**	**4.37**	**2.76**	**2.08**	**3.65**
Sweden	< 5	.	.	.	.	.	.
Eastern Europe and Former Yugoslavia	< 5	.	.	.	.	.	.
Global North and West	7	0.81	0.39	1.68	0.77	0.37	1.60
Global South and East	5	1.34	0.56	3.24	1.09	0.45	2.63
Liver							
Finnish‐born	3247	ref.	.	.	.	.	.
All migrants	41	0.97	0.72	1.33	0.90	0.66	1.23
Former USSR	13	0.69	0.40	1.17	0.62	0.36	1.10
Sweden	< 5	.	.	.	.	.	.
Eastern Europe and Former Yugoslavia	< 5	.	.	.	.	.	.
Global North and West	13	1.16	0.68	1.95	1.13	0.67	1.91
Global South and East	12	**2.00**	**1.10**	**3.61**	1.77	0.98	3.20
Pancreas							
Finnish‐born	5307	ref.	.	.	.	.	.
All migrants	54	0.80	0.61	1.03	**0.76**	**0.58**	**0.99**
Former USSR	25	0.82	0.56	1.20	0.77	0.52	1.12
Sweden	5	0.84	0.35	2.04	0.85	0.35	2.06
Eastern Europe and Former Yugoslavia	< 5	.	.	.	.	.	.
Global North and West	12	0.66	0.38	1.17	0.65	0.37	1.15
Global South and East	11	1.16	0.65	2.06	1.07	0.60	1.91
Prostate							
Finnish‐born	13,287	ref.	.	.	.	.	.
All migrants	129	0.85	0.71	1.00	**0.78**	**0.66**	**0.93**
Former USSR	67	0.94	0.75	1.19	0.84	0.66	1.06
Sweden	14	1.02	0.61	1.70	1.04	0.62	1.73
Eastern Europe and Former Yugoslavia	7	0.84	0.40	1.76	0.78	0.37	1.62
Global North and West	31	0.74	0.52	1.04	0.71	0.50	1.00
Global South and East	10	0.58	0.30	1.12	**0.51**	**0.26**	**0.99**
Urinary tract							
Finnish‐born	5147	ref.	.	.	.	.	.
All migrants	53	0.85	0.64	1.13	0.78	0.59	1.03
Former USSR	23	0.80	0.51	1.24	0.70	0.45	1.09
Sweden	11	**2.05**	**1.12**	**3.74**	**2.09**	**1.14**	**3.81**
Eastern Europe and Former Yugoslavia	< 5	.	.	.	.	.	.
Global North and West	14	0.84	0.50	1.42	0.80	0.48	1.36
Global South and East	< 5	.	.	.	.	.	.
Lymphoma and leukemia							
Finnish‐born	7210	ref.	.	.	.	.	.
All migrants	69	**0.78**	**0.62**	**0.99**	**0.73**	**0.58**	**0.93**
Former USSR	36	0.89	0.64	1.23	0.82	0.59	1.14
Sweden	7	0.87	0.41	1.84	0.88	0.42	1.86
Eastern Europe and Former Yugoslavia	< 5	.	.	.	.	.	.
Global North and West	18	0.76	0.48	1.21	0.74	0.47	1.17
Global South and East	5	0.44	0.18	1.05	**0.40**	**0.17**	**0.97**

*Note:* Model 1: Age at death, calendar year, region of residence in Finland; Model 2: Model 1 + annual personal disposable income. Statistically significant IRRs in bold (*p* ≤ 0.05). “.” indicates < 5 observations.

In men's lung cancer, Model 1 showed no statistically significant differences. However, after controlling for income in Model 2, a mortality advantage emerged in all migrants (IRR: 0.77, 95% CI: 0.67–0.89), with the most pronounced advantage observed for migrants from the Global South and East (IRR: 0.46, 95% CI: 0.28–0.75). Additionally, men born in the Former USSR and in the Global North and West exhibited a similar tendency but without reaching statistical significance.

In stomach cancer, men from the former USSR showed a mortality disadvantage in both models (Model 1, IRR: 3.32, 95% CI: 2.52–4.37; Model 2, IRR: 2.76, 95% CI: 2.08–3.65). In pancreatic cancer, Model 1 showed no statistically significant differences, but after accounting for income in Model 2, the difference became more pronounced and statistically significant for all migrants (IRR: 0.76, 95% CI: 0.58–0.99).

In liver cancer, men from the Global South and East exhibited a mortality disadvantage, which was attenuated after adjustment for income in Model 2 (Model 1, IRR: 2.00, 95% CI: 1.10–3.61, Model 2, IRR: 1.77, 95% CI: 0.98–3.20). In cancers of the urinary tract, men from Sweden showed elevated mortality risk in both models (Model 1, IRR: 2.05, 95% CI: 1.12–3.74, Model 2, IRR: 2.09, 95% CI: 1.14–3.81).

For prostate cancer, Model 1 showed a tendency toward mortality advantage for all migrants (*p =* 0.055), and with income adjustment in Model 2 the finding became stronger and statistically significant (IRR: 0.78, 95% CI: 0.66–0.93). The advantage was most pronounced for men from the Global South and East (IRR: 0.51, 95% CI: 0.26–0.99). Also, men from the Global North and West showed a similar tendency (*p* = 0.052).

For lymphoma and leukemia, a mortality advantage was observed for migrant men in both models (Model 1: IRR: 0.78, 95% CI: 0.62–0.99; Model 2: IRR: 0.73, 95% CI: 0.58–0.93). Among subgroups, only men from the Global South and East showed a statistically significantly lower mortality in Model 2 (Model 2, IRR: 0.40, 95% CI: 0.17–0.97). For colorectal cancer, no significant differences were observed between the groups of men.

## 4 Discussion

We examined cancer mortality among older migrant and non‐migrant populations in Finland. In addition, we explored the role of socio‐economic status (SES), measured as annual personal disposable income, in the migrant versus nonmigrant cancer mortality differences. Our findings contribute to the understanding of the “healthy migrant effect” in old age, which suggests that in high‐income countries, migrants often experience better health outcomes than the majority population, but the difference tapers off in old age [8]. Against the notion of tapering off, we showed that the overall cancer mortality advantage for migrants was evident also in old age. However, this advantage varied by the cancer site, sex, and region of origin. In general, men exhibited higher cancer death rates than women in both migrant and Finnish‐born groups. However, during the study period (2002–2020), cancer mortality among men declined, while rates among women remained relatively stable.

Our cancer‐site‐specific analyses revealed a lung cancer mortality advantage among both migrant men and women. Additionally, a mortality advantage was observed for men in pancreatic cancer, prostate cancer, and in lymphomas and leukemia and for women in genital cancers. We observed a mortality *disadvantage* in stomach cancer for migrants from the Former USSR countries, in liver cancer for men from the Global South and East, and in cancers of the urinary tract for men born in Sweden.

Excess risk in stomach cancer mortality was found for both migrant men and women from the Former USSR countries. Similar findings in earlier studies on younger migrants' gastric cancer have been linked to higher prevalence rates of *Helicobacter pylori* infection, a major risk factor for stomach cancer [45]. *H. pylori* infection is typically acquired in childhood and persists for life. Globally, the Former USSR region has among the highest *H. pylori* infection rates, which may explain the elevated stomach cancer mortality in this group [46]. In our analysis, adjustment for personal income somewhat attenuated the association, suggesting that SES differences may partially contribute to this disadvantage. Thus, our research provides new evidence that this mortality disadvantage persists into old age among migrants and may be partly attributed to SES. As *H. pylori* infection is treatable, targeted screening and eradication in high‐risk groups could help to reduce stomach cancer risk.

Next, we found a mortality disadvantage in liver cancer among men from the Global South and East, which disappeared after adjustment for SES, indicating that differences in SES largely explained this excess risk. Liver cancer mortality is often associated with hepatitis B virus (HBV) infection, which is more prevalent in African and Asian regions [47]. SES plays a role too, as individuals with lower SES are more vulnerable to limited healthcare access and related disparities also in high‐income countries. Reducing HBV prevalence and related mortality requires systematic screening and improved access to healthcare for at‐risk groups, including migrants from the Global South and East [47].

Men born in Sweden exhibited higher mortality for cancers of the urinary tract even after SES adjustment, suggesting that other factors contribute to this disparity. Recent research comparing bladder cancer incidence in Sweden, Finland, and Denmark suggests that the use of snuff (smokeless tobacco) may explain the stable bladder cancer rates in Sweden, in contrast to the continued decline observed in Finland and Denmark [48]. Other risk factors for urinary tract cancer include regular smoking, alcohol consumption, high body weight, and occupational exposures [49]. These findings suggest a need for targeted screening for high‐risk groups, alongside counseling on smoking and smokeless tobacco cessation.

We also examined the relationship between income differences and mortality rates. After controlling for income differences, an overall cancer mortality advantage was strengthened for both migrant men and women. This suppression effect [50], where the inclusion of a control variable magnified the coefficient of interest instead of reducing it, suggests that migrants' lower income obscures the full extent of their cancer mortality advantage.

When examining different cancer sites, a clear SES suppression pattern was found in lung, pancreas, and prostate cancer mortality among men. These cancer types are common in high‐income countries and are largely related to lifestyle factors, such as tobacco smoking, poor diet and high alcohol intake even though genetics and environmental exposures play a role too [51, 52, 53]. These findings may reflect persistent socio‐economic inequalities in health‐related lifestyles in Finland [33] and align with the healthy migrant effect, suggesting that due to selection and certain lifestyle benefits, migrants tend to be healthier than the majority populations in high‐income countries. The complex relation of SES with this advantage should be further studied.

Migrant women exhibited a lung cancer mortality advantage and, unlike in men, this advantage was not influenced by income differences. This might reflect a sex‐SES interaction, which can influence behaviors such as smoking. Our findings are in line with previous research, which has shown that in the former USSR region, social disadvantage is a risk factor for smoking especially for men [54].

Similarly, SES did not account for the observed mortality advantage in men for lymphomas and leukemia, suggesting that this benefit may be influenced by other factors, such as genetic predisposition and environmental exposure, which are central to the etiology of these cancers [55, 56]. However, the mortality advantage in lymphomas and leukemia was not observed in women. Leukemia and lymphoma are more common in men, and sex differences in biological responses, immune function, or even environmental exposures may contribute to this disparity [57]. Additionally, access to healthcare, lifestyle factors, and treatment disparities could influence outcomes differently for women [57].

For female genital cancers, the mortality advantage persisted across both models and was slightly more pronounced after accounting for SES. Genetics play a role in the etiology of these cancers, but differences in reproductive behaviors, parity patterns, diet, smoking and alcohol consumption may explain some of this advantage [58]. For women from the Former USSR the mortality advantage became significant only after adjusting for income, suggesting that socioeconomic factors may obscure the advantage. Earlier research has found that Russian‐born women in Finland participated less in cervical cancer screenings than locally born women [27]. Our findings highlight the need to improve access to and uptake of cancer screening specifically among women with lower socioeconomic status. Cervical cancer is the most common gynecological cancer in Global North and West countries, whereas endometrial cancer predominates in the Global South and East [59]. Future studies including larger migrant populations could allow for more detailed site‐specific analyses.

Finally, the previously observed breast cancer mortality advantage among non‐Western migrant women in Finland [13] was not clearly seen in our study for those aged 70 and above, as their rates did not differ significantly from Finnish‐born women. This may reflect differences in disease and survival between pre‐ and postmenopausal breast cancers. In postmenopausal breast cancer, tumors are typically slower‐growing and less aggressive than in younger women with fast‐growing, more aggressive tumors [60].

The observed mortality disadvantages and socioeconomic disparities highlight the need for enhanced and targeted cancer screening among migrants from high‐risk areas and for efforts to reduce socioeconomic disparities in preventive cancer care. In Finland, cancer screening rates are among the highest in the EU; however, participation is lower among individuals from lower socioeconomic and migrant backgrounds [27, 33]. Migrants face multiple barriers to accessing healthcare and are less likely to be diagnosed at an early stage, which is often associated with poorer cancer survival [15]. However, the evidence remains mixed and varies by country of origin, country of residence, and cancer site, with some studies indicating better survival among migrants for specific cancers compared to locally born people [16, 17, 18]. More studies and systematic reviews of cancer survival are needed to clarify these inconsistencies and to better understand the mechanisms underlying differences in cancer outcomes among migrants and nonmigrants especially in older age groups.

The migrant mortality advantage presents a paradox, as migrants often exhibit higher levels of morbidity [19]. Over‐coverage (unregistered emigration leading to individuals remaining recorded as alive in the host country population register) has been shown not to fully account for the observed all‐cause migrant mortality advantage [8]. However, the accuracy of cause‐specific mortality statistics warrants further scrutiny. Migrants are overrepresented in ill‐defined causes of death [20, 39], and these inaccuracies may bias results particularly when dealing with limited case numbers. Thus, both methodological and substantive factors must be considered when interpreting the migrant mortality advantage across different causes of death.

## 5 Strengths and Limitations

A major strength of this study is the use of a comprehensive population register spanning over 18 years, offering robust longitudinal data to analyze long‐term trends and patterns in cancer mortality among migrants and the nonmigrant population. The extensive national coverage and large sample size of the study enhance the statistical power of the analysis. In addition to Finland, the results may be applied to other countries with sufficiently similar demographic and healthcare profiles. However, the small sample sizes in some migrant groups and especially among the oldest old limit statistical inference. Limited case numbers required us to analyze some cancers in broader categories, such as female genital cancers, which restricted site‐specific analyses. Grouping was necessary, however, to ensure sufficient statistical power and comparability across migrant subgroups. Additionally, the register lacks data on the length of stay in Finland and age at migration as well as information on lifestyles and habits, all of which could be important factors influencing cancer incidence and warrant further research [61]. The Finnish Cause of Death Register is considered reliable and suitable for epidemiological research [36, 37]. Nevertheless, it may still contain inaccuracies that should be considered when interpreting the results. In particular, among older individuals the recorded underlying cause of death may be less reliable than in younger age groups due to multimorbidity and overall deterioration of health, which can increase diagnostic uncertainty [62]. Also, the accuracy of the information on remigration and migrants' overrepresentation in ill‐defined causes of death may bias the estimates [8].

## 6 Conclusion

This population register study provided new insights into cancer mortality among older migrants. It found a cancer mortality advantage for migrants in several cancer sites, with significant variations across sexes, regions of origin and SES. Notably, a migrant mortality disadvantage was observed in stomach cancer, driven by high mortality rates among individuals born in former USSR countries. In addition, a mortality disadvantage was observed for liver cancer among men from the Global South and East, and for cancers of the urinary tract among men born in Sweden. These findings highlight the need for targeted screening and culturally tailored information to improve adherence to prevention and early detection recommendations.

## Author Contributions

**Laura Kemppainen:** conceptualization, methodology, investigation, writing – original draft, writing – review and editing, visualization, formal analysis, software, data curation. **Teemu Kemppainen:** conceptualization, methodology, investigation, writing – review and editing. **Jani Raitanen:** conceptualization, methodology, data curation, writing – review and editing. **Linda Enroth:** conceptualization, writing – review and editing. **Pauliina Halonen:** conceptualization, writing – review and editing. **Marja Jylhä:** conceptualization, resources, writing – review and editing, funding acquisition. **Anne Kouvonen:** conceptualization, resources, writing – review and editing, funding acquisition. **Jutta Pulkki:** conceptualization, resources, writing – review and editing, funding acquisition.

## Ethics Statement

This study is a register‐based study utilizing individual‐level data obtained through official Finnish health and social data registers. Permission to use the data was granted to the COCTEL project by the respective register administrators. The COCTEL study plan was approved by the Pirkanmaa Hospital District Ethics Committee. As the study involves secondary use of health data without direct contact with individuals, and all analyses were performed on pseudonymized data within a secure environment, no separate informed consent from participants was required.

## Conflicts of Interest

The authors declare no conflicts of interest.

## Supporting information


**Figure S1:** Map showing the regions of origin of study participants based on country of birth.

**Table S1:** Descriptive statistics on the risk population (corrected to reflect start‐of‐the‐year situation).

## Data Availability

The data that support the findings of this study are available from the FinData Social and Health Data Permit Authority. Restrictions apply to the availability of these data, which were used under license for this study. These data are not available for public access or sharing.
