# Supplementary figures and images for: Overall and Site‐Specific Cancer Mortality Among Older Migrants and Nonmigrants in Finland: A Population Register Study on All Deaths, 2002–2020
Source: Cancer Med. 2025 Nov 23;14(22):e71380. doi: 10.1002/cam4.71380 (PMC12640617; doi:10.1002/cam4.71380)

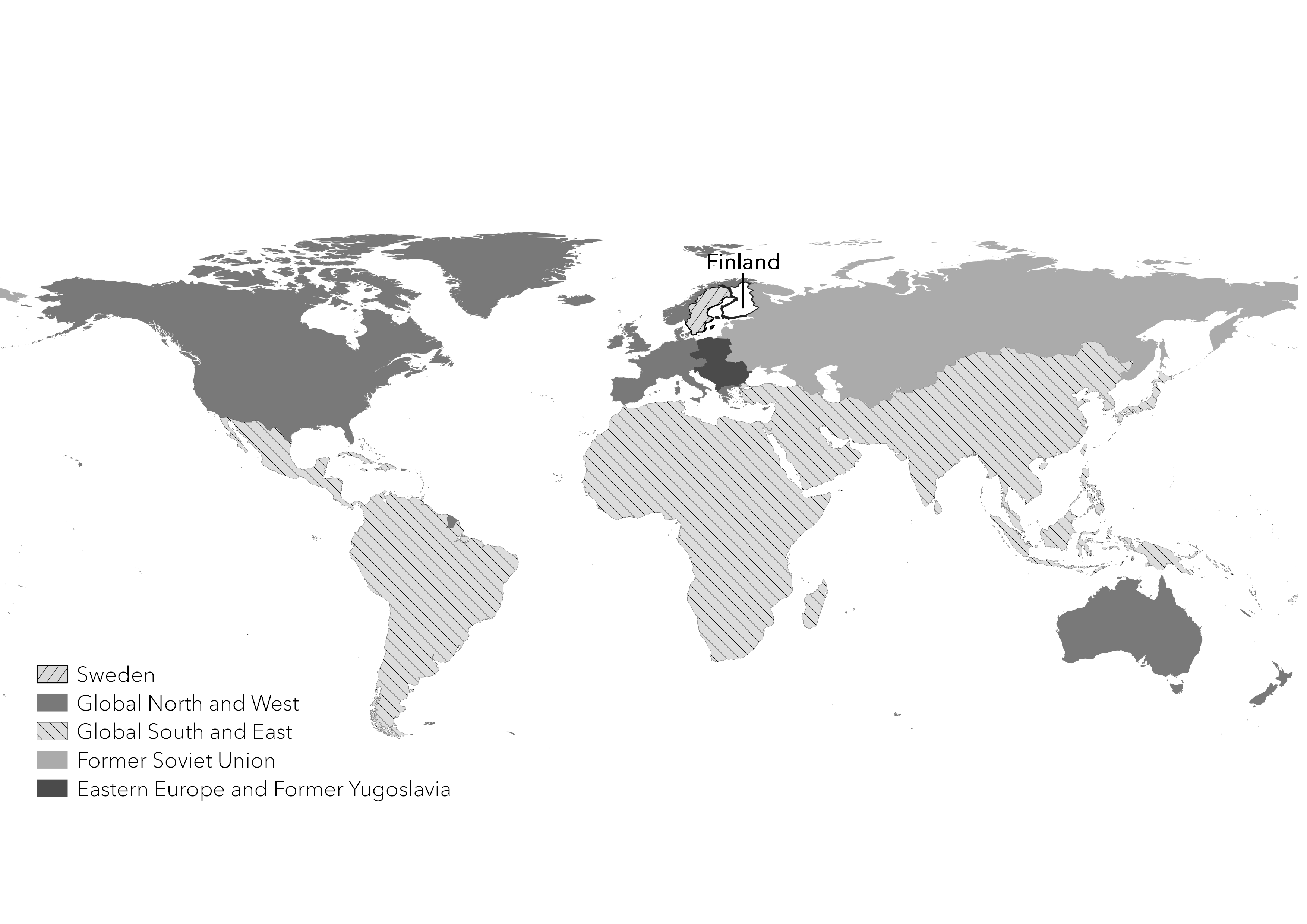

Supplement: Supplementary file 1 — Figure S1: Map showing the regions of origin of study participants based on country of birth. [file CAM4-14-e71380-s001.png]
