# Supplementary material for: Overall and Site‐Specific Cancer Mortality Among Older Migrants and Nonmigrants in Finland: A Population Register Study on All Deaths, 2002–2020
Source: Cancer Med. 2025 Nov 23;14(22):e71380. doi: 10.1002/cam4.71380 (PMC12640617; doi:10.1002/cam4.71380)
Supplement: Supplementary file 2 — Table S1: Descriptive statistics on the risk population (corrected to reflect start‐of‐the‐year situation). [file CAM4-14-e71380-s002.docx]

| **Supplementary Table S1. Descriptive statistics on the risk population (corrected to reflect start-of-the-year situation)** | | | | | | | | | | | | | | | | | | |  |
| --- | --- | --- | --- | --- | --- | --- | --- | --- | --- | --- | --- | --- | --- | --- | --- | --- | --- | --- | --- |
|  |  | Finnish-born | | All migrants | | Former USSR | | Sweden | | Former Yugoslavia and Eastern Europe | | Global North and West | | Global South and East | | Missing country of birth | | Total |  |
|  |  | **n** | **%** | **n** | **%** | **n** | **%** | **n** | **%** | **n** | **%** | **n** | **%** | **n** | **%** | **n** | **%** | **n** | **%** |
| Total |  | 13 767 397 | 98.6 | 175 626 | 1.3 | 104 784 | 0.8 | 12 455 | 0.1 | 8 617 | 0.1 | 31 499 | 0.2 | 18 271 | 0.1 | 17 314 | 0.1 | 13 960 337 | 100.0 |
| Sex | Male | 5 499 037 | 39.9 | 68 982 | 39.3 | 30 466 | 29.1 | 6 235 | 50.1 | 4 233 | 49.1 | 18 340 | 58.2 | 9 708 | 53.1 | 5 815 | 33.6 | 5 573 834 | 39.9 |
|  | Female | 8 268 360 | 60.1 | 106 644 | 60.7 | 74 318 | 70.9 | 6 220 | 49.9 | 4 384 | 50.9 | 13 159 | 41.8 | 8 563 | 46.9 | 11 499 | 66.4 | 8 386 503 | 60.1 |
| Age at death | 70–78 | 7 785 224 | 56.5 | 104 766 | 59.7 | 58 094 | 55.4 | 9 082 | 72.9 | 6 063 | 70.4 | 18 269 | 58.0 | 13 258 | 72.6 | 7 074 | 40.9 | 7 897 064 | 56.6 |
|  | 79–84 | 3 426 274 | 24.9 | 41 168 | 23.4 | 27 374 | 26.1 | 1 830 | 14.7 | 1 652 | 19.2 | 7 033 | 22.3 | 3 279 | 17.9 | 4 246 | 24.5 | 3 471 688 | 24.9 |
|  | 85–89 | 1 663 749 | 12.1 | 19 049 | 10.8 | 12 595 | 12.0 | 914 | 7.3 | 588 | 6.8 | 3 737 | 11.9 | 1 215 | 6.6 | 3 130 | 18.1 | 1 685 928 | 12.1 |
|  | 90+ | 892 150 | 6.5 | 10 643 | 6.1 | 6 721 | 6.4 | 629 | 5.1 | 314 | 3.6 | 2 460 | 7.8 | 519 | 2.8 | 2 864 | 16.5 | 905 657 | 6.5 |
| Personal annual disposable money income | |  |  |  |  |  |  |  |  |  |  |  |  |  |  |  |  |  |  |
|  | Lowest quartile | 3 093 048 | 22.5 | 83 184 | 47.4 | 59 364 | 56.7 | 1 582 | 12.7 | 3 687 | 42.8 | 10 448 | 33.2 | 8 103 | 44.3 | 5 595 | 32.3 | 3 181 827 | 22.8 |
|  | Second quartile | 3 456 584 | 25.1 | 36 928 | 21.0 | 23 305 | 22.2 | 2 127 | 17.1 | 2 012 | 23.3 | 4 899 | 15.6 | 4 585 | 25.1 | 3 904 | 22.5 | 3 497 416 | 25.1 |
|  | Third quartile | 3 292 699 | 23.9 | 25 808 | 14.7 | 13 677 | 13.1 | 2 816 | 22.6 | 1 265 | 14.7 | 4 764 | 15.1 | 3 286 | 18.0 | 3 469 | 20.0 | 3 321 976 | 23.8 |
|  | Highest quartile | 3 925 056 | 28.5 | 29 706 | 16.9 | 8 438 | 8.1 | 5 930 | 47.6 | 1 653 | 19.2 | 11 388 | 36.2 | 2 297 | 12.6 | 4 260 | 24.6 | 3 959 022 | 28.4 |
| Region of Residence (NUTS) | |  |  |  |  |  |  |  |  |  |  |  |  |  |  |  |  |  |  |
|  | Helsinki-Uusimaa | 3 011 389 | 21.9 | 79 605 | 45.3 | 46 785 | 44.6 | 4 491 | 36.1 | 3 506 | 40.7 | 13 564 | 43.1 | 11 259 | 61.6 | 5 578 | 32.2 | 3 096 572 | 22.2 |
|  | Western Finland | 3 792 244 | 27.5 | 28 752 | 16.4 | 15 740 | 15.0 | 1 619 | 13.0 | 1 767 | 20.5 | 7 130 | 22.6 | 2 496 | 13.7 | 4 587 | 26.5 | 3 825 583 | 27.4 |
|  | Southern Finland | 3 329 019 | 24.2 | 43 648 | 24.9 | 31 184 | 29.8 | 1 554 | 12.5 | 2 340 | 27.2 | 5 420 | 17.2 | 3 150 | 17.2 | 3 300 | 19.1 | 3 375 967 | 24.2 |
|  | Northern and Eastern Finland | 3 562 996 | 25.9 | 20 210 | 11.5 | 10 987 | 10.5 | 2 487 | 20.0 | 894 | 10.4 | 4 633 | 14.7 | 1 209 | 6.6 | 3 047 | 17.6 | 3 586 253 | 25.7 |
|  | Åland Islands | 71 739 | 0.5 | 3 411 | 1.9 | 88 | 0.1 | 2 304 | 18.5 | 110 | 1.3 | 752 | 2.4 | 157 | 0.9 | 719 | 4.2 | 75 869 | 0.5 |
